# Supplementary material for: Factors Affecting COVID-19 Outcomes in Cancer Patients: A First Report From Guy's Cancer Center in London
Source: Front Oncol. 2020 Jul 22;10:1279. doi: 10.3389/fonc.2020.01279 (PMC7396540; doi:10.3389/fonc.2020.01279)
Supplement: Supplementary file 1 [file Data_Sheet_1.docx]

**Appendix**

**Figure 1.** Directed Acyclic Graph (DAG) for the association between demographic and clinical characteristics of COVID-19 positive cancer patients and severity/death of COVID-19 (www.dagitty.net).


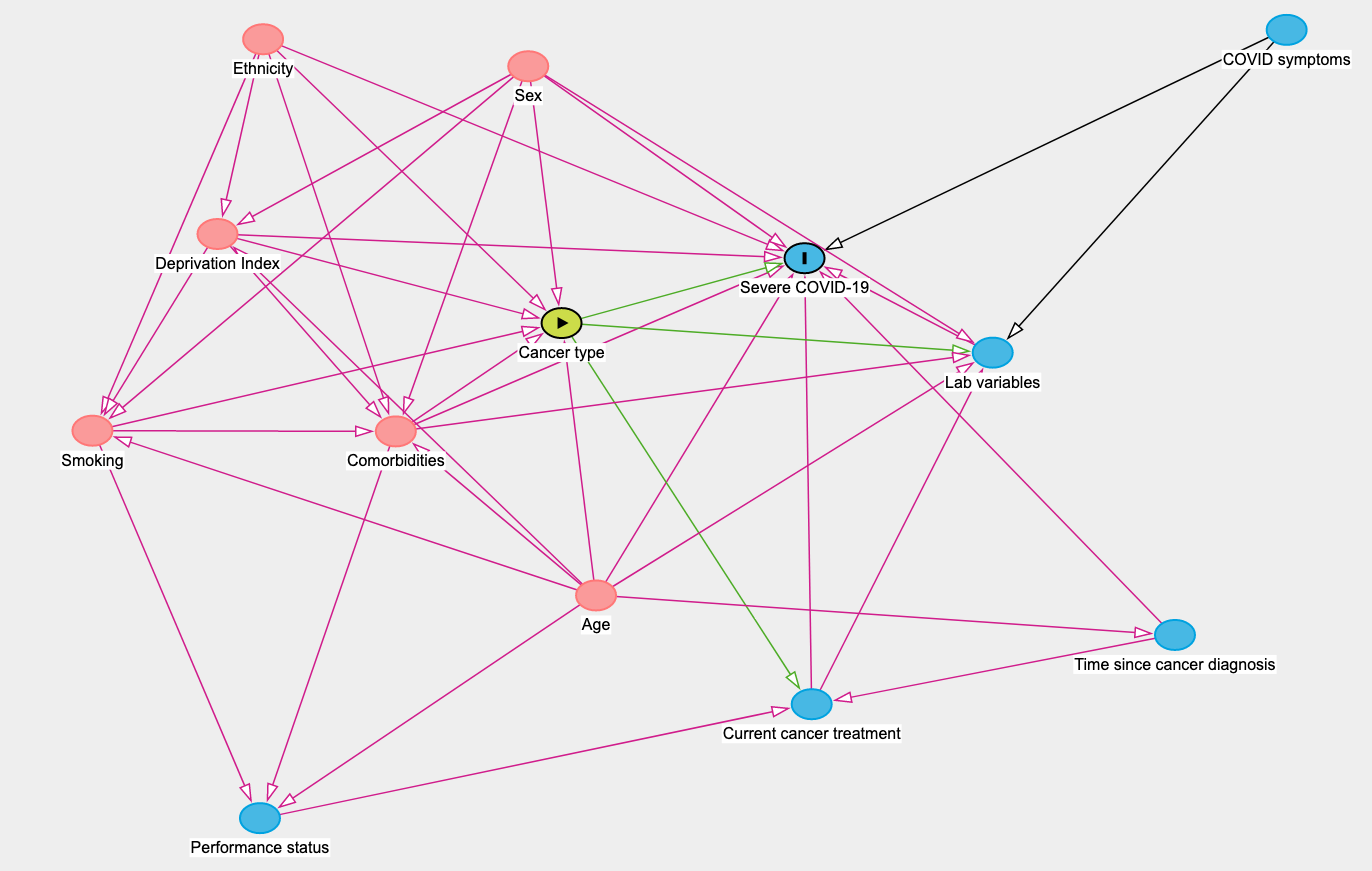


**Table 1.** Overview of minimal adjustments for the associations between demographic and clinical characteristics of COVID-19 positive cancer patients and severity/death of COVID-19 (www.dagitty.net).

| **Main exposure variable** | **Minimal adjustments** |
| --- | --- |
| Age | No adjustment is necessary to estimate the total effect of Age on severity/death of COVID-19 |
| Sex | No adjustment is necessary to estimate the total effect of Sex on severity/death of COVID-19 |
| Ethnicity | No adjustment is necessary to estimate the total effect of Ethnicity on severity/death of COVID-19 |
| Deprivation Index | Age, Ethnicity, Sex |
| Comorbidities | Age, Deprivation Index, Ethnicity, Sex, Smoking |
| Smoking | Age, Deprivation Index, Ethnicity, Sex |
| Cancer type | - Age, Comorbidities, Deprivation Index, Ethnicity, Performance status, Sex - Age, Comorbidities, Deprivation Index, Ethnicity, Sex, Smoking |
| Current cancer treatment | - Age, Cancer type, Comorbidities, Deprivation Index, Ethnicity, Sex, Time since cancer diagnosis - Age, Cancer type, Comorbidities, Smoking, Time since cancer diagnosis - Cancer type, Performance status, Time since cancer diagnosis |
| Time since cancer diagnosis | Age |
| Performance status | Age, Cancer type, Comorbidities, Deprivation Index, Ethnicity, Sex  Age, Comorbidities, Smoking |
| Lab variables | Age, COVID symptoms, Cancer type, Comorbidities, Current cancer treatment, Sex |
